# Effects of Dietary *Mallotus oblongifolius* Ultrafine Powder Supplementation on Quality of Pork from Hainan Pigs During the Late Fattening Period

**DOI:** 10.3390/vetsci12020173

**Published:** 2025-02-14

**Authors:** Yali Xie, Jilun Meng, Ruiping Sun, Jie Liu, Quanwei Liu, Yangkun Ou, Qi Qi, Xiang Li, Yan Zhang, Jingli Yuan, Manping Xing, Zhe Chao, Guiping Zhao, Limin Wei

**Affiliations:** 1 Hainan Key Laboratory of Tropical Animal Breeding and Epidemic Research, Institute of Animal Husbandry & Veterinary Research, Hainan Academy of Agricultural Sciences, Haikou 570100, China; xieyali182@126.com (Y.X.); ruiping937@126.com (R.S.); jieliu2303@163.com (J.L.); lqw502@126.com (Q.L.); 15079542301@163.com (Y.O.); qq77edu@163.com (Q.Q.); lixiang3343@163.com (X.L.); zy79818_0@163.com (Y.Z.); 13250732023@163.com (J.Y.); xingmp11920@163.com (M.X.); chaozhe@hnaas.org.cn (Z.C.); 2 Xianghu Laboratory, Hangzhou 311231, China; mengjilun@hotmail.com; 3 Sanya Institute, Hainan Academy of Agricultural Sciences (Hainan Experimental Animal Research Center), Sanya 572000, China; zhaoguiping@caas.cn

**Keywords:** MOUP, Hainan pigs, pork quality, tenderness, antioxidant, amino acids

## Abstract

The genus *Mallotus oblongifolius* (MO), a member of the Euphorbia family, exhibits a predominant distribution in Hainan Island and has been proven to possess diverse medicinal attributes. Research indicates that ultramicro-grinding fully exposes the active ingredients of *Mallotus oblongifolius*, enhancing bioavailability and efficacy, compared to before. Our study investigates the effects of ultrafine powder of *Mallotus oblongifolius* (MOUP) on Hainan pigs. A total of sixty-four healthy castrated pigs (ternary hybrid pigs, Duroc × Duroc × Tunchang) with comparable initial body weight (BW, 68.06 ± 1.03 kg, 150 days old) were allocated randomly into four groups: the control group (CONT), the antibiotic group (ANTI), the 0.1% MOUP group (PT1), and the 0.5% MOUP group (PT2). There were four replicate pens per treatment with four pigs per pen. The pre-test lasted for 7 days and the formal test lasted for 70 days. The CONT group was fed the basal diet, the ANTI group was fed the basal diet supplemented with 300 mg/kg colistin sulfate, the PT1 group was fed the basal diet supplemented with 0.1% MOUP, and the PT2 group was fed the basal diet supplemented with 0.5% MOUP. The findings of our study indicate that the inclusion of colistin sulfate and MOUP in the diet did not have any significant impact on the production performance or carcass indicators of Hainan pigs compared to the CONT group. However, it is noteworthy that the addition of MOUP to the diet resulted in a significant improvement in the lightness, tenderness, muscle fiber morphology, amino acid composition, and antioxidant activity of the longissimus dorsi muscle, particularly in the PT2 group, compared to the CONT group. In conclusion, the present study has demonstrated that the inclusion of MOUP in the dietary regimen yields enhancements in the meat quality of Hainan pigs, particularly when supplemented at a concentration of 0.5%.

## Simple Summary

Research indicates that ultramicro-grinding fully exposes the active ingredients of *Mallotus oblongifolius*, enhancing bioavailability and efficacy. Our study investigates the effects of ultrafine powder of *Mallotus oblongifolius* (MOUP) on Hainan pigs. A total of sixty-four healthy castrated pigs with comparable initial body weight were allocated randomly into four groups: the control group (CONT), the antibiotic group (ANTI), the 0.1% MOUP group (PT1), and the 0.5% MOUP group (PT2). There were four replicate pens per treatment with four pigs per pen. The findings of our study indicate that the inclusion of colistin sulfate and MOUP in the diet did not have any significant impact on the production performance or carcass indicators of Hainan pigs compared to the CONT group. However, it is noteworthy that the addition of MOUP to the diet resulted in a significant improvement in the tenderness, muscle fiber morphology, amino acid composition, and antioxidant activity of the longissimus dorsi muscle, particularly in the PT2 group, compared to the CONT group. In conclusion, the present study has demonstrated that the inclusion of MOUP in the dietary regimen yields enhancements in the meat quality of Hainan pigs, particularly when supplemented at a concentration of 0.5%.

## 1. Introduction

Since the 1950s, antibiotics have been added to animal feed for the purpose of improving the nutritional status of animals and promoting growth [1]. However, the abuse of antibiotics has led to the emergence of drug resistance [2], which is a threat to human and animal health and the environment. Many countries have banned low-dose antibiotics as growth promoters in livestock feed, accelerating research into suitable natural alternatives with similar or better beneficial effects. As consumers pay more and more attention to food quality and safety, the market needs to provide higher quality pork to meet the needs of consumers, and at the same time, improving the quality of meat through nutritional adjustment or changing the feed composition is one of the hot spots of pig nutrition research [3].

*Mallotus oblongifolius* is a genus of Euphorbia, mainly distributed in Hainan Island. *Mallotus oblongifolius* is rich in various nutrients, carbohydrates, fat, crude protein, and offers high feeding value [4]. The total free amino acids account for 0.2%, mainly containing proline, alanine, asparagine, glutamine, and so on [5]. The leaves of *Mallotus oblongifolius* are consumed as herbal tea for clearing heat, detoxification, anti-oxidation, and digestive aid, as well as being a popular health drink [6,7,8]. The main components of *Mallotus oblongifolius* include polysaccharides, polyphenols, saponins, and pigments [7,9], which contribute to the health benefits of *Mallotus oblongifolius*. It was found that with the reduction in particle size, the polysaccharide content in the powder was increased, and the polyphenols were decreased [10,11]. Polysaccharide has the biological activities of lowering blood sugar, reducing blood lipids, anti-oxidation, improving intestinal flora, and so on. Ultrafine grinding technology emerged in the 1980s; by the use of mechanical and fluid forces, the material particles are ground to a particle size of 1~10 μm using fine grinding technology [10]. Compared with coarse powder, micro powder products have uniform texture, strong fluidity and adsorption, huge specific surface area and porosity, strong chemical reactivity, high solubility, and other physical and chemical properties [12,13]. After ultramicro-grinding, the active ingredients of *Mallotus oblongifolius* are fully exposed to improve bioavailability and enhance efficacy [11]. Research suggests MO has beneficial components and potential antioxidant and resistance roles. However, its effects on pigs remain unexplored. Thus, we included the ultrafine powder derived from *Mallotus oblongifolius* (MOUP) in the feed to study its impact on the growth and meat quality of Hainan pigs during the late fattening stage.

The Hainan pig is a native endemic pig breed mainly raised on Hainan Island. It has characteristics such as heat resistance, disease resistance, and good meat characteristics, and is highly favored by consumers. Nevertheless, Hainan pigs exhibit an extended feeding cycle, sluggish growth, and suboptimal feed conversion efficiency (FCR), particularly during the latter phase of fattening.

Therefore, the objective of this study was to evaluate the effects of dietary addition of MOUP on growth performance and meat quality of Hainan pigs.

## 2. Materials and Methods

### 2.1. Preparation of MOUP

*Mallotus oblongifolius* was purchased from Dongmen Farmers’ Market, Qiongshan District, Haikou City. The leaves of one-year-old *Mallotus oblongifolius* were air-dried. An ultramicro pulverizer (LWF-6B1, Jinan Longwei Pharmaceutical Equipment Co., Ltd., Jinan, China) was used to grinds the material into powder, which was then filtered via a 300-mesh screen.

### 2.2. Animal and Experimental Design

A total of sixty-four healthy castrated pigs (ternary hybrid pigs, Duroc × Duroc × Tunchang) with comparable initial body weight (BW, 68.06 ± 1.03 kg, 150 days old) were allocated randomly into four groups: the control group (CONT), the antibiotic group (ANTI), the 0.1% MOUP (1 g MOUP per Kg of feed) group (PT1), and the 0.5% MOUP (5 g MOUP per Kg of feed) group (PT2). There were four replicate pens per treatment with four pigs per pen. The CONT group was given the basal diet (Table 1) based on the nutrient requirements for swine (NRC, 2012), the ANTI group was fed the basal diet supplemented with 300 mg/kg colistin sulfate (180122757, Best Biological technology institute Co., Ltd., Changsha, China), the PT1 group was fed the basal diet supplemented with 0.1% MOUP, and the PT2 group was fed the basal diet supplemented with 0.5% MOUP. The pre-test lasted for 7 days and the formal test lasted for 70 days. The temperature of the pig house was maintained at 25–30 °C and the humidity at 60~70%. Pigs were given ad libitum access to water. During the period, the experimental pigs were fed three times a day in the morning, mid-day, and evening, and their feed intake was recorded. Disinfection and immunization were carried out according to the routine procedures. Euthanasia, necropsy, tissue sample collection, and processing pigs were euthanized with a lethal overdose of euthanal (euthanasia solution, pentobarbital sodium, and phenytoin sodium). Experimental pigs were purchased from Tunchang Tianzhihong Ecological Agriculture and Animal Husbandry Co., Ltd., Tunchang, China. The animal test was approved by the Animal Conservation and Use Committee of Hainan Academy of Agricultural Sciences (HNSYY20230203).

### 2.3. Slaughter and Sample Collection

At the end of the experiment period, the pigs were fasted for 12 h, and individually weighed. Pig slaughtering followed the slaughter slab procedures [14]. Average daily gain (ADG), average daily feed intake (ADFI), and feed conversion efficiency (FCR) were calculated based on body weight and feed intake. Blood was collected from the anterior vena cava and the serum was prepared by tilting at room temperature for 30 min, centrifugation at 3000 r/min for 15 min, and stored in a −20° refrigerator for test. Six pigs from each treatment were randomly selected and slaughtered (at least 1 in each replicate). After slaughtering the pigs, the head, hooves, tail, and offal were completely removed and the hot carcass weight was recorded to calculate the slaughter rate. The longissimus thoracis (LT) muscles from the left carcass side between the 9th and 10th ribs were collected immediately after slaughter and stored at −80 °C until analysis.

### 2.4. Carcass Characteristics and Meat Quality

The mean backfat was measured on the first rib, last rib, and last lumbar vertebrae in the midline using a sliding caliper. The left side of the carcass was split at the 10th rib to determine the 10th-rib backfat thickness and longissimus muscle area. Based on the National Pork Producers Council, pH and flesh color CIELAB values (L*, a* and b*) of LT were determined at 45 min postmortem. A portable pH meter (FE28, METTLER TOLEDO, Shanghai, China) and chromatic aberration meter (TS7700, Sanen Shi, Shenzhen, China) were used. To determine drip loss, the meat core was weighed and tied with cotton thread (diameter 25 mm), then suspended in a sealed triangle bottle without contact to store at 4 °C for 24 h before weighing. Shear forces were determined as follows: Approximately 250 g LT per pig, refrigerated at 4 °C for 48 h, was boiled in 80 °C water to an internal temperature of 70 °C, then cooled to 25 °C. A muscle tenderness instrument (C-LM3B, Northeast Agricultural University, Heilongjiang, China) was used to cut 10 cylindrical cores (1 cm in diameter × 1 m in length) of a mature LT sample perpendicular to the direction of the muscle fibers. The average peak shear force in Newtons (N) was recorded.

### 2.5. Histochemical Staining

After removing the grease and fascia from the surface, 2 cm longissimus dorsi muscle of the slaughtered pig was placed in a centrifuge tube filled with 4% paraformaldehyde solution to completely soak and fix the shape of muscle fibers. Wuhan Xyever Biotechnology Co, Ltd. (Wuhan, China). was commissioned to perform HE stain sealing. Five samples were randomly chosen from each group, and three images from each sample were analyzed using Image J software (1.30v).

### 2.6. Antioxidant Indices of Muscle

The total antioxidant capacity (A015-2-1), superoxide dismutase (A001-3-2), glutathione peroxidase (A005-1-2), and malondialdehyde (A003-1-2) contents of muscle were determined using the kit produced by Nanjing Jiangcheng Institute of Bioengineering according to the kit instructions.

### 2.7. Distribution of Amino Acids in Longissimus Pectoralis Muscle

The amino acid content in muscle was detected according to the determination method of amino acid in food (GB 5009.124-2016, China). The meat sample was freeze-dried and crushed for detection. The sample of 1 g longissimus dorsi muscle was placed in a 10 mL HCl (6 mol/L) hydrolyzed tube, sealed with nitrogen for three times after freezing, and placed in a constant temperature drying oven. Hydrolysis at 110 °C for 22 h to 24 h was performed. After cooling, the hydrolysate was transferred and 0.02 mol/L HCl was used in a 50 mL flask. After mixing, 1 mL solution was transferred to a 15 mL test tube and evaporated in a 65 °C water bath until dry. The sample was dissolved with 2 mL HCl (0.02 mol/L), filtered (0.22 μm), transferred to the instrument sample bottle, and determined by an amino acid automatic analyzer L-8900. Guangzhou Jinzhi Detection Technology Co., Ltd. (Guangzhou, China) was commissioned to carry out the analysis.

### 2.8. Statistical Analysis

The data collected from all experiments (*n* = 6) were initially summarized using Excel software, followed by analysis using one-way ANOVA conducted with SPSS 20 software (International Business Machines Co., Armonk, NY, USA). All the table data are expressed as means ± SD, and the figure data are expressed as means ± SEM. *p*-values < 0.05 were considered significant.

## 3. Results

### 3.1. The Effect of MOUP on the Growth Performance of Hainan Pigs During the Fattening Stage

Based on the data presented in Table 2, there was no statistically significant disparity in the initial body weight among the CONT group, the ANTI group, the PT1 group, and the PT2 group. The addition of antibiotics and MOUP does not exert any significant influence on the final weight, ADG, ADFI, and FCR of Hainan pigs (*p* > 0.05).

### 3.2. The Effect of MOUP on Carcass Performance of Hainan Pigs During the Fattening Stage

According to the data presented in Table 3, the inclusion of MOUP and 300 mg/kg colistin sulfate did not yield a statistically significant impact on carcass performance when compared to the CONT group (*p* > 0.05).

### 3.3. The Effect of MOUP on Pork Quality of Hainan Pig During the Fattening Stage

As can be seen from Table 4, the lightness of the PT1 and PT2 groups was significantly increased compared with the ANTI group (*p* < 0.05), but had no significant difference compared with the CONT group (*p* > 0.05). The pH on the 24th hour of the PT1 and PT2 groups exhibited a significant increase when compared to the ANTI group (*p* < 0.05). However, no significant difference was observed between the PT1 and PT2 groups and the CONT group (*p* > 0.05). The PT2 group exhibited a significant decrease in shear force compared to the CONT group (*p* < 0.05). However, there was no significant difference in shear force between the PT2 group and the ANTI group or the PT1 group (*p* > 0.05). The inclusion of MOUP did not yield any statistically significant variations in meat quality parameters, including redness, yellowness, pH at the 45th minute, drip loss, and loin-eye area (*p* > 0.05).

### 3.4. Effects of MOUP on Morphology of Longissimus Dorsi Muscle of Hainan Pigs in the Fattening Stage

**Figure 1:**
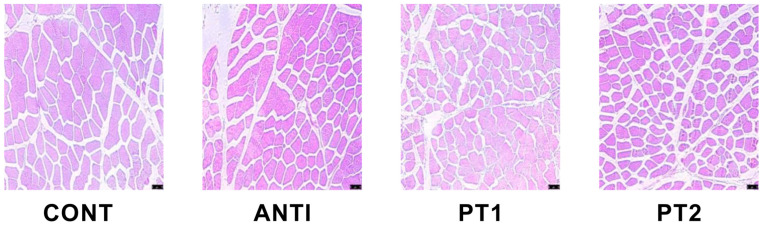
The impact of MOUP on the elongation of the dorsal region of Hainan pigs during the final stage of fattening. Scale bar: 75 μm.

As shown in Figure 1 and Table 5, compared with the CONT group and ANTI group, the PT1 and PT2 groups showed significantly reduced diameter (*p* < 0.01) and average area of muscle fibers (*p* < 0.05).

### 3.5. Effects of MOUP on Antioxidant Activity of Longissimus Dorsi Muscle of Hainan Pigs in the Fattening Stage

As can be seen from Table 6, compared with the CONT group, the total antioxidant capacity (T-AOC) content of the PT2 group was significantly increased (*p* < 0.05), but had no significant difference from the ANTI and PT1 groups. Compared with the CONT group and ANTI group, the glutathione peroxidase (GSH-Px) content of the PT1 group and PT2 group was significantly increased (*p* < 0.05). There was a lack of statistically significant disparity observed in the concentrations of malondialdehyde (MAD) and superoxide dismutase (SOD) among the four groups.

### 3.6. Influence of MOUP on Amino Acid Content in Longissimus Dorsi Muscle of Hainan Pigs in the Late Fattening Period

We measured the content of amino acids to evaluate the nutritional value and flavor of the Longissimus dorsi muscle. According to the data analysis in Table 7, compared with the CONT group, alanine, arginine, leucine, phenylpropyl, tyrosine, and total amino acid (TAA) of the PT1 group and PT2 group were significantly increased (*p* < 0.05). In addition, the glycine content of the PT2 group was significantly higher than that of the CONT group (*p* < 0.05). Compared with the CONT group, there was no significant change in amino acid content in the ANTI group (*p* > 0.05). Moreover, the proline content in longissimus dorsi muscle were significantly decreased in the PT1 group and PT2 group (*p* < 0.05). There were no significant changes observed in the essential amino acids (EAAs) and free amino acids (FAAs) across the four groups, although there was an increase in content noted in the PT1 and PT2 groups.

## 4. Discussion

The economic value of livestock and poultry is closely associated with the quality of meat, and consumers’ purchasing decisions are directly influenced by the color of the meat. The pH value and meat color serve as crucial indicators of meat freshness. The pH 45 min value provides insights into the acidity and alkalinity of the pork itself, while the pH 24 h value indicates the rate at which the pork’s acidity and alkalinity decline [15]. Typically, within 45 min of slaughter, the muscle pH is lowered from 6.3 to 6.7, and the muscle color and hydration rate remain within normal levels [16]. However, when the pH is lowered to 5.5, particularly under high-temperature conditions, the muscle undergoes protein deterioration, exudation increase, color paleness, and a change in texture, resulting in the formation of white muscle (PSE) meat [17]. In the present study, the inclusion of 0.1% and 0.5% MOUP in the diet resulted in an improvement in the L* value and a decrease in the change in pH value. Notably, the inclusion of 0.5% MOUP in the diet also led to a reduction in shear force, which is an important indicator of muscle tenderness. Shear force is the current evaluation index of tenderness; the greater the shear force, the older the meat, and vice versa [18]. The potential explanation for the observed phenomenon could be attributed to the ability of tea polyphenols to augment the antioxidant activity of cell membrane lipids, thereby safeguarding their structural integrity and stability. This protective effect mitigates the degradation of muscle proteins, leading to a gradual decline in pH levels and a reduction in the surface reflectance of meat, which contribute to the enhancement of muscle lightness [19]. Previous studies also support our results; the supplementation of tea polyphenol compound additive in the diet of finishing pigs increased the a* value and decreased the shear force [20]. Moreover, dietary supplementation of 400 mg/kg and 500 mg/kg tea polyphenols in finishing pigs significantly decreased muscle shear force and increased the pH, L* value, a* value, and b* value [21]. Unlike our findings, tea polyphenol can enhance the a* and b* values of meat, likely because of its different primary functional components compared to MOUP.

To conduct a more comprehensive examination of the impact of MOUP on muscular tissues, we conducted an analysis of the morphological characteristics of muscle fibers. Muscle fibers make up 70% to 90% of muscle volume, and their morphology and characteristics are key determinants of muscle mass and pork quality [22]. In our study, feeding 0.1% and 0.5% MOUP significantly reduced the diameter and the mean area of muscle fiber. In addition, the extract derived from MO, known for its antioxidant properties, exhibited a protective effect against ethanol-induced acute gastric mucosa injury in rats [7]. This effect was attributed to the extract’s ability to enhance the content of SOD, CAT, and GSH-Px enzymes, while concurrently reducing the levels of ROS, MDA, and MPO content [23]. The current investigation revealed that the application of 0.5% MOUP resulted in a significant augmentation of T-AOC and GSH-Px levels. Consequently, MOUP exhibits advantageous properties in terms of enhancing the antioxidant capacity of the longissimus dorsi muscle.

Amino acids are the basic unit of protein composition, from the perspective of nutrition; the type, content, and proportion of amino acids in protein determine the nutritional value of pork [24,25]. Therefore, the content of amino acids in the carcass has a great relationship with the flavor and quality of meat [26,27]. The presence of flavor amino acids, namely aspartic acid, glutamic acid, proline, glycine, and serine, has a direct impact on the sensory perception of pork flavor [28]. On the other hand, lysine, threonine, tryptophan, and sarcosine influence the physicochemical characteristics of muscle tissue and overall meat quality, consequently influencing the overall quality of pork [27]. *Mallotus oblongifolius* is abundant in various amino acids, such as alanine, proline, asparagine, glutamine, and other indispensable amino acids [5]. In the present study, it was observed that the inclusion of MOUP in the diet resulted in a notable augmentation of the overall amino acid composition in the longest dorsal muscle. Our study revealed that incorporating MOUP into the diet significantly changes the amino acid profile of the longest dorsal muscle. Adding 0.1% and 0.5% MOUP increased arginine, alanine, leucine, phenylalanine, tyrosine, and TAA, while decreasing proline [25]. In particular, 0.5% MOUP also boosted glycine levels. Notably, the dosage of 0.5% exhibited the most pronounced impact. The aforementioned evidence demonstrates that the utilization of MOUP, a superfine powder, has the potential to enhance muscle flavor and augment muscle nutrient content.

Growth performance is an important manifestation of the economic value of pigs. Improving growth performance of pigs and shortening the time of putting out pigs can increase economic income. Our results showed that dietary supplementation of 0.1% and 0.5% MOUP had no significant effect on the growth performance of Hainan pigs in the late fattening stage. Articles have indicated that the inclusion of green tea or tea in the diet does not exert any discernible impact on the production performance of pigs during the latter phases of fattening [29,30]. Additionally, it has been documented that the inclusion of 6% green tea powder results in a decrease in ADFI, while the incorporation of 5% tea powder leads to a reduction in ADG [31]. The reduction in polyphenol and alkaloid content in the bitter tea following ultramicro-grinding may not have had an impact on the feed intake of pigs. Moreover, the presence of anti-nutritional factors in tea powder can diminish the digestibility of dietary protein in pigs and consequently influence their weight gain [32].

Slaughter performance is the main indicator used to measure the economic benefits of breeding. Our results showed that the supplementation of MOUP did not affect the hot carcass weight, slaughter rate, backfat thickness, fat mass, abdominal fat rate, or lean mass of pigs. However, a recent study has demonstrated that the inclusion of 2% tea powder in pig feed yields a notable increase in lean meat percentage, as well as a significant augmentation in eye muscle area and hind leg ratio [33]. Furthermore, another investigation has revealed that the incorporation of 4% tea powder in the diet of Tibetan fragrant pigs leads to the lowest backfat thickness and fat percentage, accompanied by the highest lean meat percentage [30]. According to the report, the inclusion of tea by-products in the diet of fattening pigs resulted in a linear decrease in backfat thickness as the quantity of tea by-products increased [34]. Previous studies have found that tea polyphenols can affect fat deposition by regulating lipid metabolism [35,36]. The findings of this research exhibit incongruity with the aforementioned studies, potentially attributable to the comparatively minimal dosage employed or the specific type of tea utilized [10].

## 5. Conclusions

In general, the inclusion of 0.1% and 0.5% MOUP in the diet of Hainan pigs does not exert any discernible impact on their growth performance and carcass indicators. However, it does yield a notable enhancement in meat quality. Furthermore, the addition of 0.5% MOUP to the diet exhibits a more pronounced effect on pork quality compared to the inclusion of 0.1% MUOP. Our subsequent investigations have revealed that the influence of MOUP supplementation on pork quality primarily manifests in three key aspects: muscle tenderness, antioxidant capacity, and the concentration of muscle amino acids. Next, we will examine the key active ingredients in MOUP, like flavonoids and volatile oils, to understand their chemical properties and content variations.

## Figures and Tables

**Table 1:** Composition and nutrient levels of basal diet.

Items	Content %
Ingredients	
Corn	71.60
Soybean meal	10.80
wheat bran	13.60
Premix ^1^	4.00
Total	100.00
Nutrient levels ^2^	
DE/(MJ/Kg)	13.03
CP	13.00
Ca	0.48
TP	0.48
Leu	1.06
Met	0.23
Lys	0.56
Thr	0.49
Try	0.16

^1^ Premix, provided per kg of the complete diet: vitamin A, 3000IU; vitamin D3, 1100IU; vitamin E, 587IU; vitamin K3, 12.5 mg; vitamin B2, 240 mg; vitamin B6, 52.5 mg; niacin, 725 mg; calcium pantothenate, 437 mg; copper, 150 mg; sodium, 2.5 mg; ferrous, 1200 mg; manganese, 300 mg; zinc, 1200 mg; potassium, 6 mg. ^2^ DE: digestive energy, CP: crude protein, TP: total phosphorus, Leu: leucine, Met: methionine, Lys: lysine, Thr, threonine, Try: tryptophan.

**Table 2:** The effects of MOUP on the growth performance of Hainan pigs during the fattening stage.

Items ^1^	CONT	ANTI	PT1	PT2	*p*-Value
Initial weight, kg	67.97 ± 0.95	67.59 ± 1.80	68.16 ± 0.74	67.81 ± 0.81	0.914
Final weight, kg	117.94 ± 2.12	116.88 ± 0.54	114.96 ± 0.73	117.72 ± 3.87	0.390
ADG, g/d	713.84 ± 41.53	704.02 ± 30.49	670.83 ± 21.53	712.95 ± 66.50	0.595
ADFI, g/d	3202.01 ± 12.39	3185.53 ± 17.80	3179.17 ± 11.25	3201.87 ± 34.56	0.604
FCR	4.50 ± 0.25	4.53 ± 0.18	4.55 ± 0.41	4.52 ± 0.43	0.997

^1^ ADG = average daily gain, ADFI = average daily feed intake, FCR = feed conversion ratio. CONT = basal feed group, ANTI = basal diet supplemented with 300 mg/kg colistin sulfate, PT1 = basal diet supplemented with 0.1% MOUP, PT2 = basal diet supplemented with 0.5% MOUP.

**Table 3:** The effect of MOUP on carcass performance of Hainan pigs during the fattening stage.

Items	CONT	ANTI	PT1	PT2	*p*-Value
Hot carcass weight, kg	87.90 ± 3.83	89.56 ± 4.13	89.6 ± 4.24	89.28 ± 4.55	0.881
Slaughter rate, %	74.31 ± 1.92	74.09 ± 2.65	76.23 ± 0.67	74.78 ± 1.52	0.286
Backfat thickness, mm	2.31 ± 0.28	2.63 ± 0.13	2.57 ± 0.27	2.38 ± 0.41	0.294
Fat mass, %	21.47 ± 4.07	25.14 ± 1.36	24.99 ± 1.95	22.87 ± 2.25	0.116
Abdominal fat rate, %	6.65 ± 1.32	6.78 ± 0.53	5.53 ± 1.33	4.97 ± 0.91	0.069
Lean mass, %	52.76 ± 4.96	51.31 ± 1.99	50.43 ± 3.44	51.37 ± 2.23	0.749

**Table 4:** The effects of MOUP on pork quality of Hainan pig during the fattening stage.

Items	CONT	ANTI	PT1	PT2	*p*-Value
Lightness L*	43.54 ^ab^ ± 1.07	42.10 ^b^ ± 0.53	45.46 ^a^ ± 2.22	45.42 ^a^ ± 1.91	0.023
Redness a*	2.06 ± 1.72	2.13 ± 1.18	2.06 ± 0.73	2.23 ± 1.78	0.997
Yellowness b*	10.92 ± 0.32	10.27 ± 0.47	10.05 ± 0.48	10.51 ± 1.27	0.314
pH 45 min	6.41 ± 0.34	6.31 ± 0.35	6.31 ± 0.38	6.14 ± 0.26	0.651
pH 24 h	5.53 ^ab^ ± 0.08	5.40 ^b^ ± 0.18	5.73 ^a^ ± 0.26	5.68 ^a^ ± 0.06	0.046
Shear force	41.81 ^a^ ± 4.93	37.58 ^ab^ ± 2.77	36.89 ^ab^ ± 2.64	31.84 ^b^ ± 5.17	0.027
Drip loss	3.66 ± 1.36	3.63 ± 0.79	3.54 ± 1.36	3.37 ± 0.55	0.978
Loin-eye area, cm^2^	41.84 ± 5.61	38.69 ± 4.97	40.21 ± 13.19	36.17 ± 3.64	0.703

Note: Different lowercase letters indicate significant difference between different experimental groups (*p* < 0.05).

**Table 5:** Influence of MOUP on morphology of longissimus dorsi muscle.

Items	CONT	ANTI	PT1	PT2	*p*-Value
Diameter, μm	92.38 ^a^ ± 8.73	93.11 ^a^ ± 7.16	68.59 ^b^ ± 7.72	69.25 ^b^ ± 6.71	0.001
Average area, mm^2^	0.0058 ^a^ ± 0.0013	0.0057 ^ab^ ± 0.0009	0.0041 ^b^ ± 0.0011	0.0045 ^b^ ± 0.0012	0.017

Note: Different lowercase letters indicate significant difference between different experimental groups (*p* < 0.05).

**Table 6:** The effects of MOUP on antioxidant activity of longissimus dorsi muscle.

Items	CONT	ANTI	PT1	PT2	*p*-Value
T-AOC mM	0.04 ± 0.024 ^b^	0.058 ± 0.012 ^ab^	0.068 ± 0.018 ^ab^	0.082 ± 0.021 ^a^	0.044
SOD U/ml	42.89 ± 4.11	43.93 ± 4.44	39.08 ± 7.71	34.02 ± 6.95	0.089
GSH-Px	15.73 ± 9.4 ^b^	13.3 ± 4.94 ^b^	36.47 ± 7.97 ^a^	38.88 ± 18.51 ^a^	0.003
MDA nmol/mL	0.114 ± 0.041	0.122 ± 0.044	0.092 ± 0.033	0.094 ± 0.031	0.535

Note: Different lowercase letters indicate significant difference between different experimental groups (*p* < 0.05).

**Table 7:** Effects of MOUP on amino acids of longissimus dorsi muscle (g/100 g).

Items [15]	CONT	ANTI	PT1	PT2	*p*-Value
Alanine	1.17 ^c^ ± 0.02	1.20 ^bc^ ± 0.05	1.26 ^ab^ ± 0.05	1.30 ^a^ ± 0.06	0.014
Arginine	1.29 ^c^ ± 0.01	1.34 ^bc^ ± 0.07	1.44 ^a^ ± 0.06	1.42 ^ab^ ± 0.08	0.009
Glycine	0.90 ^bc^ ± 0.03	0.88 ^c^ ± 0.03	0.93 ^ab^ ± 0.03	0.95 ^a^ ± 0.05	0.020
Leucine	1.69 ^b^ ± 0.02	1.78 ^ab^ ± 0.08	1.84 ^a^ ± 0.07	1.88 ^a^ ± 0.09	0.013
Phenylalanine	0.83 ^b^ ± 0.01	0.88 ^ab^ ± 0.04	0.90 ^a^ ± 0.03	0.93 ^a^ ± 0.04	0.011
Tyrosine	0.73 ^b^ ± 0.01	0.77 ^ab^ ± 0.04	0.81 ^a^ ± 0.03	0.81 ^a^ ± 0.05	0.020
Proline	0.65 ^a^ ± 0.02	0.62 ^ab^ ± 0.04	0.59 ^b^ ± 0.04	0.58 ^b^ ± 0.04	0.017
Aspartate	2.02 ± 0.11	2.05 ± 0.11	2.08 ± 0.12	2.07 ± 0.14	0.830
Threonine	0.99 ± 0.06	1.02 ± 0.06	1.03 ± 0.04	1.03 ± 0.07	0.482
Serine	0.84 ± 0.05	0.87 ± 0.05	0.86 ± 0.05	0.87 ± 0.06	0.764
Glutamate	3.27 ± 0.23	3.42 ± 0.27	3.24 ± 0.22	3.23 ± 0.27	0.503
Valine	1.09 ± 0.05	1.11 ± 0.07	1.1 ± 0.05	1.1 ± 0.07	0.924
Methionine	0.57 ± 0.03	0.59 ± 0.03	0.62 ± 0.03	0.61 ± 0.05	0.253
Isoleucine	1.04 ± 0.06	1.07 ± 0.06	1.08 ± 0.06	1.07 ± 0.08	0.76
Lysine	2.13 ± 0.12	2.19 ± 0.13	2.21 ± 0.14	2.2 ± 0.16	0.778
histidine	1.04 ± 0.05	1.06 ± 0.06	1.06 ± 0.04	1.05 ± 0.07	0.885
TAA ^1^	19.75 ^b^ ± 0.24	20.93 ^ab^ ± 0.99	21.26 ^a^ ± 0.78	21.63 ^a^ ± 1.02	0.032
EAA ^2^	8.93 ± 0.39	9.14 ± 0.45	9.53 ± 0.33	9.44 ± 0.61	0.184
FAA ^3^	9.01 ± 0.50	9.11 ± 0.52	9.32 ± 0.37	9.33 ± 0.57	0.652

^1^ TAA: total amino acids. ^2^ EAA: essential amino acids. ^3^ FAA: free amino acids. Note: Different lowercase letters indicate significant difference between different experimental groups (*p* < 0.05).

## Data Availability

The datasets used and/or analyzed during the current study are available from the corresponding author upon reasonable request.
